# Neonatal bilateral adrenal hemorrhage and adrenal insufficiency accompanied by Subgaleal hematoma: a case report with brief review of literature

**DOI:** 10.1186/s12887-022-03314-1

**Published:** 2022-05-05

**Authors:** Golnaz Ghazizadeh Esslami, Atousa Moienafshar

**Affiliations:** 1 Department of Emergency, Pediatrics Center of Excellence, Children’s Medical Center, Tehran University of Medical Sciences, Tehran, Iran; 2 Department of Newborn Nursery, Neonates, and Pediatrics, Ziaeian Hospital, Tehran University of Medical Sciences, Tehran, Iran; 3 Department of Family Medicine, Ziaeian Hospital, Tehran University of Medical Sciences, Tehran, Iran; 4 Pediatrics Department, school of medicine, Ziaeian Hospital, Tehran University of Medical Sciences, Tehran, Iran

**Keywords:** Vaginal delivery, Neonatal adrenal hemorrhage, Adrenal insufficiency, Ultrasound monitoring, Hormonal therapy, Subgaleal hemorrhage (SGH)

## Abstract

**Background:**

Neonatal adrenal hemorrhage (NAH) is an almost infrequent phenomenon (0.2–0.55%). Mechanical compression and alterations of venous pressure during delivery are considered the most probable explanations. Approximately 10% of the cases might have bilateral involvement. Clinical symptoms include abdominal mass, poor feeding, vomiting, prolonged jaundice, and anemia. Subgaleal hemorrhage (SGH) is one of the most clinically remarkable and potentially hazardous postnatal cranial injuries.

**Case presentation:**

An early-term Iranian male neonate who was born through spontaneous vaginal delivery and experienced shoulder dystocia was diagnosed with bilateral NAH leading to adrenal insufficiency requiring glucocorticoid and mineralocorticoid supplementation. The SGH and jaundice were other postnatal complications. Serial monthly abdominal and brain ultrasound revealed complete regression of lesions after 70 days. However, after 16 months, the neonate has been still treated with hydrocortisone and fludrocortisone for the adrenal insufficiency diagnosis. He has a lower limit weight for age; however, developmental milestones have been appropriate for age.

**Discussion and conclusion:**

Adrenal hemorrhage and SGH should be examined and looked for, particularly with proven evidence of difficult delivery and asphyxia in at-risk newborns. Clinical and ultrasound follow-up is mandatory for the assessment of hemorrhage resolution and conservative management. The early detection and treatment of adrenal insufficiency by laboratory examination is strongly recommended in bilateral cases. Furthermore, the early recognition of postnatal SGH to prevent clinical and neurological outcomes seems essential.

## Background

Neonatal adrenal hemorrhage (NAH) is an almost infrequent disorder (0.2–0.55%) [1]. The peak of incidence is during the first weeks of life [2]. It is remarkable that the adrenal glands in newborns are approximately 10–20 times bigger in size in comparison with adults relative to body weight. Nevertheless, as 50–60 arterial branches originate from 3 suprarenal arteries, an enhancement in vascularity is resulted [3, 4]. Adrenal hemorrhage most frequently (approximately 70%) appears on the right side. The right adrenal gland anatomical location which is between the liver and spine, can easily lead to its compression. Furthermore, it’s compression due to the drainage of right adrenal vein into the inferior vena cava can predispose venous pressure alterations [5, 6]. However, 10% of the cases might have bilateral involvement [1].

The etiology of adrenal hemorrhage is commonly multifactorial. Mechanical compression and alterations of venous pressure during delivery are considered the most probable causes. In hypoxia, with redistribution of blood to the central nervous system, heart, and adrenal glands, hemorrhage as a result of congestion and endothelial damage is anticipated [7]. Other factors inducing NAH include asphyxia, shock, septicemia, and preexisting coagulation disorders. Furthermore, vaginal delivery, macrosomia, and fetal acidemia are presented as the most crucial predisposing factors of NAH, according to a retrospective survey [8]. However, in a large proportion of cases, NAH cause could not be distinguished.

Term neonates are more commonly affected by NAH. It is also mainly observed in male neonates due to their higher birth weight [1]. The NAH might remain asymptomatic; however, clinical symptoms include abdominal mass, poor feeding, vomiting, prolonged jaundice, and anemia. Jaundice, as the most frequent symptom, is caused by hemolysis due to an enclosed hemorrhage [9, 10]. Discoloration, hematoma, swelling in scrotum, as well as lethargy, hypotonia and hypertension are among other symptoms [11, 12]. Although rare, hematuria could be categorized as another clinical manifestation of adrenal hemorrhage in neonates [13]. Severe unilateral cases might be associated with hypovolemic shock; however, in bilateral cases, evidences of hypoadrenocorticism are more noticeable [1].

The NAH is commonly self-limited due to the resolution and absolute regression of lesions within the 20th to 165th day of life [14]. In neonates, ultrasound modality due to portability, sensitiveness, noninvasiveness, and nonexistence of ionizing radiation takes the priority for both the preliminary screening and the follow-up assessment. Computed tomography and magnetic resonance imaging generally do not provide supplementary data, although they might be beneficial for the detection of hemorrhage and progression of hemoglobin breakdown [15].

Subgaleal hemorrhage (SGH) is one of the most clinically remarkable and potentially hazardous postnatal brain injuries [16]. As blood has the potential to accumulate abundantly in the loose connective tissue between the galea aponeurotica and the pericranium of the scalp, neonates influenced by SGH are exposed to significant bleeding, leading to serious morbidity or even mortality [17]. The SGH, most commonly detected following assisted vaginal deliveries, is rarely diagnosed after unassisted vaginal or cesarean deliveries [18]. This report presents a male neonate with bilateral adrenal hemorrhage accompanied by SGH following prolonged labor leading to adrenal insufficiency.

## Case presentation

An early-term Iranian male neonate was born through spontaneous vaginal delivery at a gestational age of 37 weeks and 4 days. The pregnancy course proceeded smoothly and without a hitch. The sole pregnancy complication was maternal hypothyroidism. Monitoring during the labor process revealed no noteworthy changes; nonetheless, right after the vaginal delivery of the head, the neonate’s anterior shoulder was entrapped inside the mother’s pelvis (shoulder dystocia). Finally, efforts to release the shoulder were effective, and the neonate was born after exerting sufficient force.

The first birth stage (i.e., full dilatation of the cervix) and second birth stage (i.e., neonate moving down through the vagina) each took 1 hour, and the third stage (i.e., delivery of the placenta) took 5 minutes. The neonate’s Apgar score was estimated at 7/10 (acrocyanosis, hypotonia, and weak cry) and 9/10 (acrocyanosis) at the 1st and 5th minutes, respectively. The case required no resuscitation, including positive pressure ventilation, at birth time. The Apgar score was improved through an oxygen blender and warmed humidifier for a few minutes (from the 1st minute to the 5th minute).

Oxygen saturation was shown about 95% by pulse oximeter without oxygen therapy at the 10th minute. The umbilical cord arterial blood gas was acceptable (pH: 7.337, PCO_2_: 43.4, PO_2_: 31, HCO_3_: 23.3, BE: − 2.8). His birth weight was 3300 g (within percentile of 25–50). The case had a birth height of 50 cm (50th percentile) and a head circumference of 34.5 cm (25th percentile). Moreover, 1 mg of intramuscular vitamin K_1_ (phytomenadione) was administered after birth. The evidence of trauma was obvious. A tense 5 × 5 cm cephalohematoma was observed in the occipitoparietal region. Diffuse petechiae, purpura, and ecchymosis were detected all around the body, particularly above the lips and in the upper limbs.

Half an hour after the delivery, the neonate was admitted to the neonatal department of Ziaeian hospital, Tehran, Iran due to hypotonia, drowsiness, and no proper sucking with the impression of neonatal asphyxia and sepsis. Laboratory studies were used to evaluate early-onset sepsis. Empiric antibiotic therapy with intravenous ampicillin and amikacin and a continuous infusion of 10% glucose solution was administered due to feeding difficulties. Blood sugar and vital signs, including arterial pressure, were within the normal limits.

Laboratory evaluations revealed normal complete blood count, electrolytes, and coagulation profile and negative C-reactive protein, blood culture, and cerebrospinal fluid culture. Nevertheless, lactate dehydrogenase (LDH) and creatine phosphokinase (CPK) were calculated above 3000 units per liter. Aspartate aminotransferase (serum glutamic-oxaloacetic transaminase) was 81, although other liver function tests appeared normal. Accordingly, hypoxic-ischemic encephalopathy grade one was strongly suggested for the patient.

Moreover, indirect hyperbilirubinemia increased gradually and was apparent on the 1st day of the newborn’s life. Therefore, intensive phototherapy started on the 1st postnatal day (total bilirubin: 9.6, direct Coombs: negative for ABO incompatibility, reticulocyte: 5.5, red blood cell morphology: normal, glucose-6-phosphate dehydrogenase: sufficient). Despite using phototherapy, neonatal jaundice quickly progressed. Therefore, phototherapy was continued up to the discharge time for about 10 days. Moreover, the neonate was evaluated for hypovolemic shock by checking blood pressure and serial hemoglobin. Simultaneous with sepsis workup and phototherapy, the physical examination of the head, including the head circumference and assessment of the location and characteristics of any swelling, was performed.

**Fig. 1:**
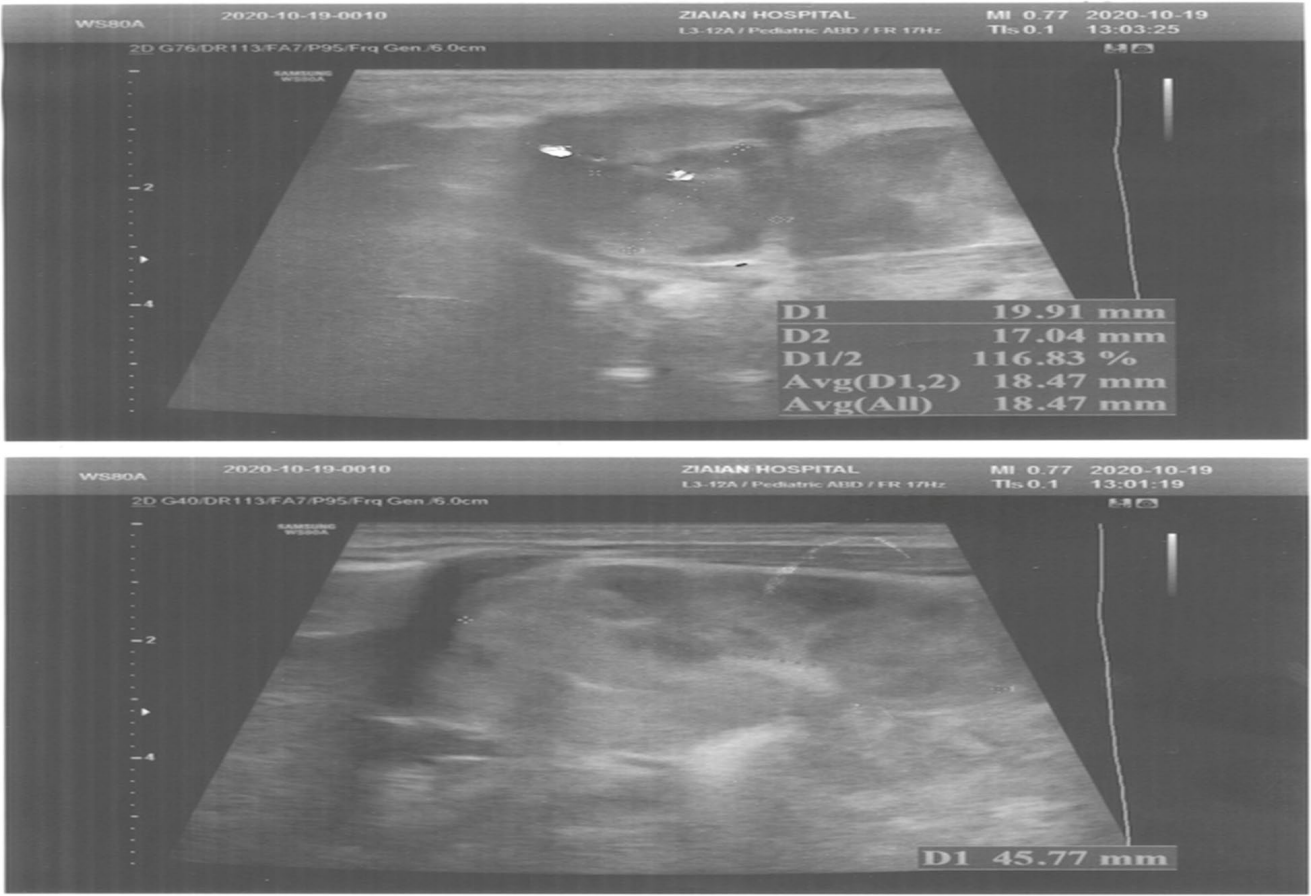
The images (17mm × 19mm right) were strongly suggestive of adrenal hemorrhage due to expert radiologist diagnosis

Within the first 24 hours, brain and abdominal ultrasounds were carried out as soon as the radiologist was available, in which the brain ultrasound appeared normal; however, the abdominal ultrasound revealed a well-circumscribed, hypoechoic, and slightly heterogenic suprarenal right lesion (Fig. 1); the images (right: 17 mm × 19 mm) were strongly suggestive of adrenal hemorrhage due to expert radiologist diagnosis (Fig. 1).

**Fig. 2:**
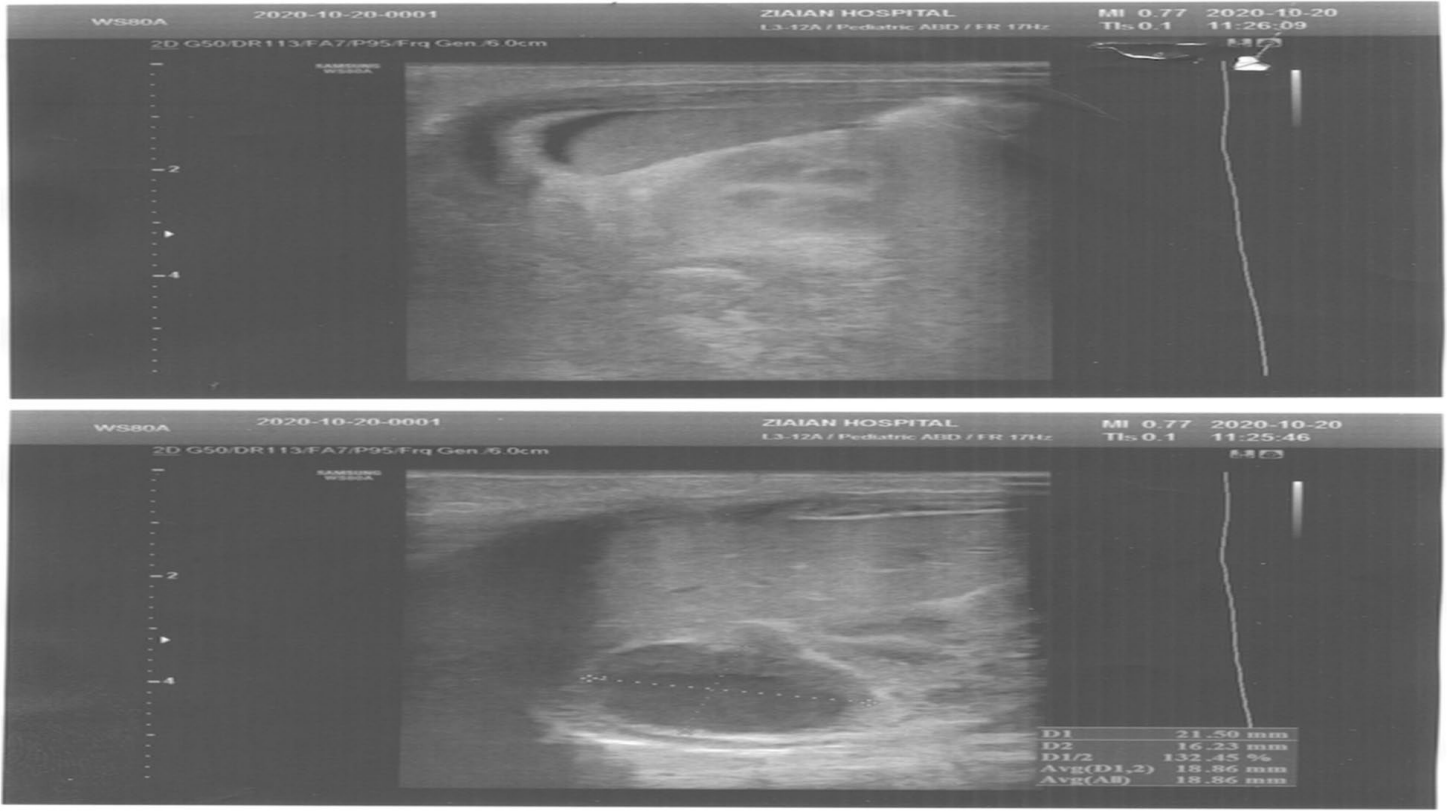
The images (16.2 mm × 21.5mm right) were mostly hypoechoic and illustrated an increase in adrenal hemorrhage size.

On the 2nd postnatal day, abdominal ultrasound was repeated by the same radiologist, and a suprarenal right lesion was detected again. The images (right: 16.2 mm × 21.5 mm) were mostly hypoechoic and showed an increase in adrenal hemorrhage size (Fig. 2).

**Fig. 3:**
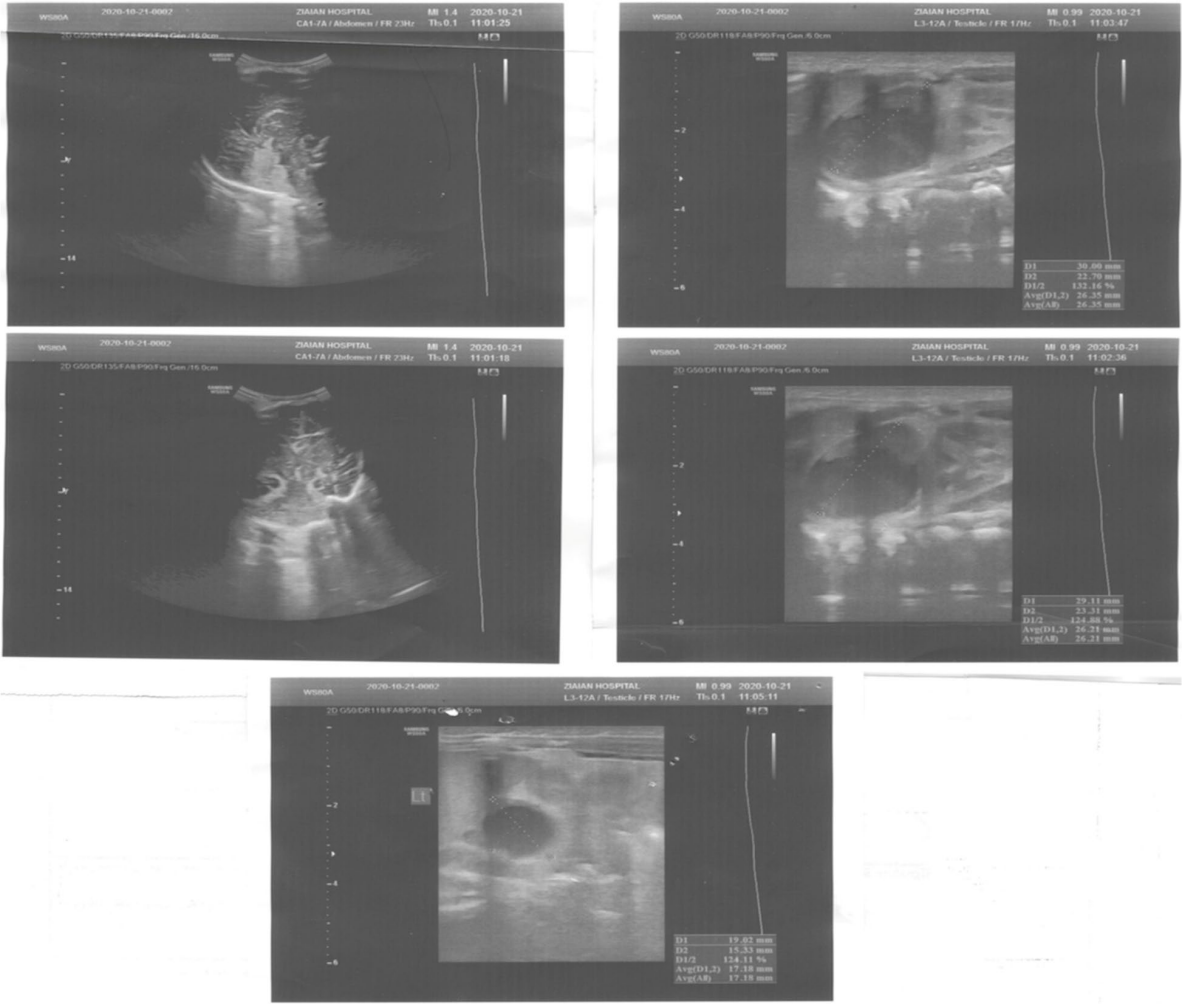
Suprarenal bilateral lesions, the images (22mm × 30mm right, 15mm × 19mm left) were mostly isoechoic-anechoic. There was a solid portion, but also fluid level, some internal echoes, and minimum turbidity with essentially normal kidneys and no foci of blood flow within the area of the NAH.

On the 3rd postnatal day, the neonate was transferred to a subspecialty center for children (Children’s Medical Center, Tehran, Iran). Brain and abdominal ultrasounds were performed again by an expert pediatric radiologist. At this time, brain ultrasound depicted SGH (hypoechoic lesion with lace-like regions, 5 × 28 mm in the occipital area) and suprarenal bilateral lesions; the images (right: 22 mm × 30 mm, left: 15 mm × 19 mm) were mainly isoechoic-anechoic (Fig. 3). There was a solid segment, in addition to fluid level, some internal echoes, and minimal turbidity accompanied by basically normal kidneys and no area of blood flow within the NAH zone. Although the blood pressure appeared normal during the admission period, the hemoglobin level dropped from 19.6 to 15.8 in the first 3 days.

On the 4th postnatal day, abdominal ultrasound displayed hypoechoic lesions without vascularity in both adrenal glands (right: 11 mm × 27 mm, left: 11 mm × 15 mm). Brain ultrasound displayed cystic germinal matrix hemorrhage (GMH) in the left caudothalamic region (2 mm × 3 mm). A neurosurgery consultation was requested in which the neonate discharge was considered unimpeded in case of consciousness, hemodynamic and hemoglobin stability, feeding tolerance, and absence of seizure. Neurology and endocrinology consultation was also held. Finally, the neonate was discharged after 11 days with normal vital signs, including blood pressure of 70/35 mmHg, a hemoglobin level of 14.8 g/dl, bilirubin level of 7.4 mg/dl (direct: 0.4), sodium level of 135 mEq/L, and potassium level of 4.2 mEq/L. Laboratory studies for neonatal jaundice workup and electrolytes were all normal.

Further investigations after discharge revealed that adrenocorticotropic hormone (ACTH) was in the normal range; however*,* the serum morning cortisol level was below the proposed lower cut-off. (2.82 μg per deciliter with a normal value of 5–25). Consequently, oral hydrocortisone with 1 mg per kilogram dosage was administered and then tapered gradually up to 70 days of age. Serial monthly abdominal ultrasound was carried out in outpatient follow-ups by the same operator indicating a complete regression of lesions after 70 days. Brain ultrasound was also performed up to the detection of no evidence of hemorrhage after 70 days.

At this stage, 8 a.m. cortisol was 21.8 μg per deciliter with a normal value of 5–25 μg; however, ACTH was significantly increased to 353 pg per milliliter with a normal upper limit of 46 pc/ml. The sodium and potassium levels of 133 and 5.2 were also detected, respectively. Since then, hydrocortisone was stepped up, and fludrocortisone was added with 0.01 mg/kg dosage. After about 3 months, ACTH, BS, and electrolytes turned normal, and hydrocortisone tapering and fludrocortisone interruption were recommended (April 2021). However, ACTH was increased in the follow-ups, and lower limit sodium level and upper limit potassium level were detected. Therefore, medication therapy for adrenal insufficiency was continued by a pediatric endocrinologist, and tapering has not been possible to date. At present, the patient is 10 kg in his 17th month (25th percentile) and is still receiving hydrocortisone (0.75 mg/kg/daily) and fludrocortisone (0.001 mg/kg/daily).

Remarkably, the neonate did not exhibit any clinical manifestations of adrenal insufficiency. Moreover, he had normal developmental milestones in 3-month, 6-month, 12-month and 17-month follow-ups. Furthermore, other laboratory tests (i.e., thyroid function test, zinc level, ferritin level, vitamin D level, and tissue transglutaminase IgA), which were evaluated due to the low percentile of weight for age, appeared within the normal range. It was also notable that the patient blood sugar and blood pressure were within the normal range during the admission and after the discharge. Table 1 shows detailed laboratory data consistent with adrenal insufficiency.

**Table 1:** Laboratory results and intervals for adrenal insufficiency

Test interval	ACTH (Up to 46 pikogram/ml)	Cortisol 8 AM (5–25 microgram/dl)	Na (135–145)	K (3.5–5.5)
one week after discharge	48.6	2.82	135	4.2
3 month later	21.8	353	133	5.2
2 month later	41.4	–	134	4.6
1 month later	123	–	136	6.2
2 month later	124	–	134	4.6
1 month later	132	7.39	137	4.7
2 month later	63.7	–	138	4

## Discussion and conclusion

The NAH prevalence range is estimated within 1.7–2.1 per 1000 births; however, the actual incidence is supposed to be higher as bleeding might remain asymptomatic [19]. Only 10% of the cases present bilaterally [20, 21]. Therefore, this case was among the infrequent cases of bilateral adrenal hemorrhage. Risk factors for NAH formation include gestational diabetes mellitus, macrosomia, perinatal asphyxia, severe sepsis, prolonged delivery, breech presentation, assisted birth with instruments, bleeding disorders, anticoagulant administration, hypothrombinemia, and child abuse [21, 22]. Any condition causing hypoxia induces shifting the blood toward the vital organs, consisting of adrenal glands. Besides, ACTH released due to stress could be harmful to the vascular endothelium and make it vulnerable to hypoxia and hemorrhage [23].

In the present case, the possible identified risk factors were vaginal delivery, prolonged and difficult labor, and suspected asphyxia. In line with the results of other studies suggesting male gender predominance, the current patient was a male neonate [11]. As previously mentioned in the introduction section, clinical manifestations are variable or even absent. Jaundice as the most frequent clinical manifestation, along with hypotonia and poor feeding, was observed in this case [9–11].

The extensive use of perinatal ultrasound has given rise to the diagnosis of an increasing proportion of suprarenal masses in newborns [24]. Adrenal hemorrhage almost arises as a homogeneous or inhomogeneous echogenic lesion with the blood clotting and clot lysis leading to variable echoes inside the mass. The central section of the adrenal bleeding commonly exhibits a pseudocyst picture that emerges as a lesion with a hypoechoic center with or without inner calcifications; such lesions progress in about 10 days after the initiation of the adrenal beeding [25]. Therefore, hypoechoic lesions with internal echo in the present study are compatible with adrenal hemorrhage diagnosis. Kidneys are recommended to be scanned in favor of renal vein thrombosis after NAH diagnosis [12]. This examination was also carried out for the present case.

Adrenal hemorrhages mostly resolve within 3 to 3.5 months with a maximum limit of 9 months in follow-up ultrasound [11, 19]. Therefore, at least 90 days should be considered for the follow-up of hemorrhage resolution. Similarly, in this patient, complete resolution occurred after 70 days. Neuroblastoma should be considered if the mass remains after this time span [26]. After the regression of hemorrhages, calcification could be observed in one-third of children, which was not apparent in this case [19]. Therefore, calcification could be a preliminary sign of neuroblastoma. Other adrenal neoplasms, congenital adrenal hyperplasia, pulmonary sequestration, and intestinal duplication should be regarded as other differential diagnoses of adrenal masses [1].

The cytokine-related suppression of ACTH or cortisol synthesis, insufficient blood supply of the adrenal gland, restricted adrenocortical reserve, or immaturity of the hypothalamic-pituitary-adrenal axis cooperate to originate adrenal insufficiency in adrenal hemorrhages. This case report confirms the literature claim that bilateral cases are more susceptible to adrenal insufficiency [1]. Consequently, glucocorticoid and mineralocorticoid supplementation were administered for this patient.

Cephalohematoma, an accumulation of blood between the skull and its periosteum, was evident in this newborn examination. Furthermore, SGH, a potentially lethal condition, was shown in ultrasound, which can clinically be distinct from cephalohematoma, as it extends more widely and crosses the suture lines [27, 28]. The SGH occurrence has been approximated to be roughly 0.4 in 1000 of normal vaginal deliveries without using instruments and 5.9 in 1000 vacuum-assisted labors [17]. The pathogenesis of neonatal SGH focuses on any trauma to the scalp. The subgaleal space is a potential space where two adjacent tissues can be separated to generate a newly formed cavity [29]. The management of SGH involves neonatal intensive care unit monitoring and serial quantification of the hemoglobin or hematocrit and head circumference, which were accomplished in this case [30].

The long-term prognosis of neonates with SGH is almost acceptable. Arterial blood pH is proved to be significantly associated with mortality which was normal in this neonate [31].

SGH and Adrenal hemorrhage should be examined and sought for in vulnerable neonates, particularly with proven evidence of difficult delivery and asphyxia. Clinical and ultrasound follow-up is mandatory for the assessment of hemorrhage resolution and conservative management. The early detection and treatment of adrenal insufficiency by laboratory examination is strongly recommended in bilateral cases. Furthermore, the early recognition of postnatal SGH to prevent clinical and neurological outcomes seems essential.

## Data Availability

No datasets were generated or analyzed during the current study.

## Abbreviations

SGH: Subgaleal hemorrhage
ACTH: Adrenocorticotropic hormone
BS: Blood Sugar
NAH: Neonatal adrenal hemorrhage
US: Ultrasound
CT: Computed tomography
MRI: Magnetic resonance
CSF: Cerebrospinal fluid
LDH: Lactic acid dehydrogenase
CPK: Creatine phosphokinase
AST: Aspartate aminotransferase
ABO: Blood types A, B, and O Blood Typing System
RBC: Red Blood Cell
G6PD: Glucose-6-phosphate dehydrogenase
